# The Role of RNA Methylation in Regulating Stem Cell Fate and Function-Focus on m^6^A

**DOI:** 10.1155/2021/8874360

**Published:** 2021-10-28

**Authors:** Weiwei Sun, Bin Zhang, Qingli Bie, Na Ma, Na Liu, Zewei Shao

**Affiliations:** ^1^Institute of Forensic Medicine and Laboratory Medicine, Jining Medical University, Jining, Shandong, China; ^2^Department of Laboratory Medicine, Affiliated Hospital of Jining Medical University, Jining, Shandong, China

## Abstract

The biological role of RNA methylation in stem cells has attracted increasing attention. Recent studies have demonstrated that RNA methylation plays a crucial role in self-renewal, differentiation, and tumorigenicity of stem cells. In this review, we focus on the biological role of RNA methylation modifications including N6-methyladenosine, 5-methylcytosine, and uridylation in embryonic stem cells, adult stem cells, induced pluripotent stem cells, and cancer stem cells, so as to provide new insights into the potential innovative treatments of cancer or other complex diseases.

## 1. Introduction

More than 150 types of RNA modifications have been identified, and they are widely distributed among various types of RNA, such as messenger RNA (mRNA), transfer RNA (tRNA), ribosomal RNA (rRNA), microRNA (miRNA), and long noncoding RNA (lncRNA) [1]. RNA methylation is one of the most crucial modifications, which mainly includes N6-methyladenosine (m^6^A), 5-methylcytosine (m^5^C), and uridylation (U-tail) [1, 2]. In general, RNA methylation has distinct functions in different types of RNA. Stem cells are a group of cells with self-renewal and multidirectional differentiation potential [3]. They are the core constituent of regenerative medicine and have been widely used in the treatment of various diseases, such as nervous system, immune system, and hematopoietic diseases [4]. Recent studies have shown that RNA methylation plays a crucial role in the self-renewal and differentiation of stem cells and tumorigenicity of cancer stem cells (CSCs) [5].

Among the RNA methylation modifications, m^6^A is one of the most abundant modifications in eukaryotes, and the epitranscriptomic detection technology has been focused on m^6^A, making it the most in-depth modification in stem cells. However, perhaps due to the lack of effective methods for detecting other RNA modification sites, the research on m^5^C and U-tail in stem cells is limited. This review summarizes the molecular mechanisms of RNA methylation and the biological functions of RNA methylation in embryonic stem cells (ESCs), adult stem cells (ASCs), induced pluripotent stem cells (iPSCs), and CSCs, with a focus on m^6^A.

## 2. RNA Methylation Modifications

RNA methylation modifications mainly include m^6^A, m^5^C, and U-tail, and their molecular mechanisms are summarized as follows.

m^6^A is one of the most abundant modifications in mRNA and lncRNA, which was first discovered in the 1970s [6, 7]. m^6^A accounts for approximately 0.1%–0.4% of adenosines and 50% of total methylated ribonucleotides in mammalian RNA [8]. The m^6^A modification is dynamically reversible and mainly occurs in RRACH (R represents A or G and H represents A, U, or C) sequences [8]. Three key factors are involved in m^6^A modification, including “writers,” “erasers,” and “readers.” “Writers” mediate the RNA methylation process, which is a methyltransferase complex composed of three core components: methyltransferase-like (METTL) 3, METTL14, and Wilms' tumor 1-associating protein (WTAP) [9, 10]. In addition, zinc finger CCCH-type containing 13 (ZC3H13), RNA-binding motif 15 (RBM15), virilizer-like m^6^A methyltransferase associated (VIRMA), Cb1 protooncogene-like 1 (CBLL1), KIAA1429, and HAKAI are involved in RNA methylation as auxiliary elements [9–11]. METTL5 has recently been identified as an RNA methyltransferase, which catalyzes m^6^A in 18S rRNA [12]. “Erasers” mediate the demethylation of RNA. Fat mass and obesity-associated protein (FTO) and alkylated DNA repair protein alkB homolog 5 (ALKBH5) are two main demethylases currently known [9]. “Readers” are responsible for “reading” the information of RNA methylation modification and participating in the translation, degradation, and other processes of downstream RNA [13]. YT521-B homology domain-containing family (YTHDF) (including YTHDF1, YTHDF2, YTHDF3, YTHDC1, and YTHDC2), the heterogeneous nuclear ribonucleoprotein (hnRNP) family (HNRNPA2B1, HNRNPC, and HNRNPG), insulin-like growth factor 2 mRNA binding protein 1~3 (IGF2BP1~3), and eukaryotic initiation factor 3 (EIF3) are m^6^A binding proteins [13–15]. Although m^6^A may block nonstandard A:G base pairing and affect RNA structure, it does not alter the coding ability or base pairing of adenine with uracil or thymine [8]. In addition, the occurrence of m^6^A may affect the expression level, translation efficiency, nuclear retention, splicing, and stability of RNA [10, 14]. m^6^A is closely related to human obesity, cancer, and other diseases and participates in the regulation of circadian rhythm, cell meiosis, cell reprogramming, stem cell proliferation, and other biological processes [6, 8, 16, 17].

In addition to m^6^A, m^5^C is another methylated modification form widely present in RNA [18, 19]. It is a crucial regulator of RNA export, ribosome assembly, translation, RNA stability, and other crucial biological processes [20–24]. m^5^C methyltransferases mainly include tRNA methyltransferase 4 (TRM4), tRNA aspartic acid methyltransferase 1 (TRDMT1), and the NOL1/NOP2/SUN domain (NSUN) family [20, 21, 25, 26]. However, enzymes capable of reversing m^5^C methylation have not been found in RNA. Although the demethylation of RNA m^5^C may be catalyzed by Tet, further verification is required [27]. ALYREF and Y-box binding protein 1 (YBX1) act as readers to specifically recognize m^5^C modification [28, 29].

U-tail, first identified in the 1970s [2], can promote the degradation of RNA, affect its processing, and change its pathway or activity [30–32]. U-tail “writers” are mainly terminal uridylyltransferases (TUTs) [2, 32, 33]. TUT4 (also known as ZCCHC11 and TENT3A) and TUT7 (ZCCHC6 or TENT3B) were identified as two important TUTases involved in mRNA degradation [32]. Whether deuridase as an “eraser” plays a regulatory role in the process of U-tail remains unclear. The 3′–5′ exonuclease DIS3L2 and Lsm1-7 complex may be the “readers” or “effectors” for selective U-tail recognition [2]. U-tail, associated with many human diseases, such as cancer and cardiac myotonic dystrophy, plays a vital role in virus immune defense and early vertebrate development [2, 30, 33].

The discovery of RNA methyl modifiers and binding proteins and their involvement in the metabolic processing of RNA suggest that RNA methylation has crucial biological functions.

## 3. m^6^A and Embryonic Stem Cells

**Figure 1:**
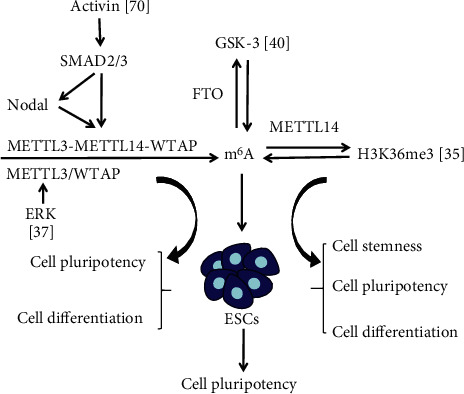
m^6^A RNA methylation interacts with H3K36me3, ERK, GSK-3, and Activin/Nodal signaling pathway to regulate ESC self-renewal, differentiation, cell stemness, and pluripotency.

The dynamic regulation of m^6^A modification is essential for the pluripotency, self-renewal, and differentiation of ESCs (Table 1).

m^6^A is the most prevalent posttranscriptional modification of mRNA and is involved in mRNA stability, splicing, translation, and other biological processes [34]. Huang et al. demonstrated that m^6^A mRNA modifications are enriched near the peaks of histone H3 trimethylation at Lys36 (H3K36me3), which is a histone marker, and are reduced when H3K36me3 is depleted in mouse ESCs [35]. METTL14, a major component of the m^6^A methyltransferase complex, can recognize and bind to H3K36me3 and then promote the cotranscriptional deposition of m^6^A. In mouse ESCs, *METTL14* knockdown and H3K36me3 loss significantly reduced m^6^A levels in transcriptome-wide and pluripotency transcripts, leading to increased cell stemness. H3K36me3 inhibits pluripotency and promotes the differentiation of mouse ESCs partially by regulating the m^6^A modifications of several key pluripotency genes, such as octamer-binding transcription factor 4 (*OCT4*), sex-determining region Y- (SRY-) box 2 (*SOX2*), and *NANOG* [35] (Figure 1). These results indicated that m^6^A RNA methylation and histone modification interact in the regulation of ESC pluripotency and differentiation. In addition, m^6^A was identified as a regulator for terminating naive pluripotency in mice to ensure timely pluripotent factor downregulation, which is necessary for proper lineage initiation and differentiation [36]. Loss of *METTL3* in preimplantation epiblasts and naive ESCs leads to near complete depletion of m^6^A on mRNA. However, *METTL3*^−/−^ ESCs still retain their naive pluripotency. Abnormal and restricted lineage initiation subsequently occurs in the postimplantation phase and results in early embryonic lethality [36]. These results are the foundation for studying the role of m^6^A in other stem cell developmental transitions and exploring other potential m^6^A functions. Sun et al. reported that extracellular regulated protein kinases- (ERK-) dependent phosphorylation of METTL3 and WTAP promotes ESC differentiation [37]. Lack of METTL3/WTAP phosphorylation reduces decay of m^6^A-labeled pluripotent factor transcripts and traps mouse ESCs in the pluripotent state [37] (Figure 1). Another m^6^A methyltransferase METTL5 has recently been shown to be important for ESC pluripotency and differentiation. Ignatova et al. reported that the absence of *METTL5* in mouse ESCs results in a decrease in global translation rate, spontaneous loss of pluripotency, and compromised differentiation potential [12]. Xing et al. also found that deletion of *METTL5* causes a dramatic differentiation defect in mouse ESCs [38]. ZC3H13, a zinc finger protein, plays a vital role in regulating RNA m^6^A methylation in ESC nuclei. ZC3H13 works together with the WTAP–VIRILIZER–HAKAI complex to promote mRNA m^6^A processing [39]. ZC3H13 is necessary for the nuclear localization of WTAP, VIRILIZER, and HAKAI. *ZC3H13* knockout in mouse ESCs remarkably reduced m^6^A levels, damaged self-renewal, and triggered differentiation [39]. The above studies suggested that these m^6^A methyltransferases play an important role in ESC differentiation and pluripotency through different regulatory mechanisms.

The RNA demethylase FTO is typically phosphorylated by glycogen synthase kinase- (GSK-) 3 and leads to polyubiquitination, which is impaired in GSK-3-knockout ESCs, resulting in increased FTO protein levels [40] (Figure 1). The m^6^A levels of pluripotency-related mRNAs Esrrb and c-Myc decreased because of the alteration of FTO protein levels [40]. The study indicated that FTO and GSK-3 interactions are involved in the regulation of stem cell pluripotency.

The YTHDF proteins have recently been proposed to perform different cellular functions. Wang et al. reported that depletion of *YTHDF3* in ESCs resulted in the loss of pluripotency with accelerated expressions of marker genes involved in the formation of three germ layers [41]. Liu et al. showed that knockout of METTL3 or YTHDC1 in mouse ESCs increases chromatin accessibility and activates transcription in an m^6^A-dependent manner [42]. Lasman et al. systematically knocked out (KO) each of the YTHDF1/2/3 readers and the three readers together (triple-KO) to analyze the effect in vitro in mouse ESCs and found that only triple-KO ESCs are not able to differentiate properly and present a prolonged mRNA half-life while no significant effect is seen in the single-KOs [43]. This suggested that there is compensation between the three YTHDF reader proteins in mouse ESCs.

Similar to the role of m^6^A mRNA modification on the fate of ESCs, m^6^A on lncRNA also affects ESC pluripotency and differentiation. Yang et al. reported that linc1281 deletion affects the differentiation of mouse ESCs, but linc1281 was not necessary for the self-renewal of mouse ESCs [44]. The m^6^A modification is significantly enriched in linc1281 transcripts. linc1281 containing the RRACU m^6^A sequence motifs could restore the differentiation capacity in linc1281-deficient mouse ESCs [44]. Mechanistically, linc1281 regulates mouse ESC pluripotency and differentiation by sequestering relevant let-7 miRNAs, which depend on m^6^A [44].

## 4. m^6^A and Adult Stem Cells

Many studies have reported the crucial biological functions of m^6^A modification in different ASCs. Here, we summarize the research results of m^6^A modification in hematopoietic stem cells (HSCs), neural stem cells (NSCs), and bone marrow mesenchymal stem cells (BMSCs) (Table 1).

### 4.1. m^6^A and HSCs

HSCs are derived from ESCs and have high self-renewal and multiple differentiation capabilities [45]. HSCs develop into mature blood cells according to certain rules under the influence of various regulatory factors in the hematopoietic microenvironment [45]. The m^6^A modification plays a key role in hematopoietic development during vertebrate embryogenesis [46].

**Figure 2:**
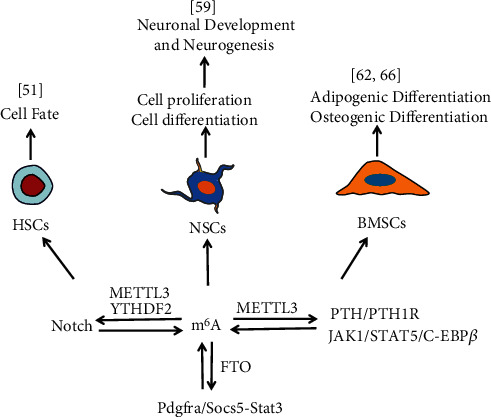
m^6^A RNA methylation interacts with multiple signaling pathways to regulate the proliferation and differentiation of different ASCs.

METTL3 is a key component of the m^6^A methyltransferase complex. *METTL3* deletion promotes cell differentiation and reduces cell proliferation in human hematopoietic stem and progenitor cells (HSPCs) [47]. By contrast, wild-type *METTL3* overexpression inhibits cell differentiation and promotes cell growth in vitro [47]. Notably, Lee et al. reported opposite results: the conditional deletion of *METTL3* in the adult hematopoietic system resulted in HSC accumulation in bone marrow, and the blockade of HSC differentiation led to a significant reduction of reconstitution potential both in vivo and in vitro [48]. However, the number and function of myeloid cells were not affected by *METTL3* deletion. The authors identified m^6^A targets in HSCs through RNA sequencing and found notable enrichment of 2073 genes related to hematopoietic differentiation, and the m^6^A modification of these genes was dependent on *METTL3* [48]. In addition, *MYC* was identified as a major functional target of m^6^A in HSCs. Further validating the difference between the two studies warrants further research, and the role of m^6^A in HSCs and progenitor cells may require individual identification. Cheng et al. uncovered that m^6^A is essential for the maintenance of HSC identity and symmetric commitment, with normal asymmetric commitment upon *METTL3* depletion [49]. Gao et al. showed that loss of *METTL3* resulted in defective fetal hematopoietic progenitor proliferation, lineage commitment, and maturation, with accumulation of immature HSPCs and resultant hematopoietic failure [50]. Zhang et al. also revealed the crucial role of m^6^A modification in the fate of HSPCs during vertebrate embryogenesis. The authors found that m^6^A peaks are obviously enriched in RRACH motifs in zebrafish, and this result is consistent with studies in mammals [51]. In *METTL3*-deficient embryos, delayed YTHDF2-mediated mRNA decay of the arterial endothelial genes notch1a and rhoca resulted in a significant decrease of m^6^A levels and blockade of HSPCs [51] (Figure 2). Because the continual activation of Notch signaling in arterial endothelial cells of *METTL3*-deficient embryos blocked the endothelial-to-hematopoietic transition, the production of the earliest HSPCs was inhibited [51]. These findings indicated that METTL3 may be a central regulator of HSPC fate.

Li et al. confirmed the role of m^6^A reader protein YTHDF2 in the maintenance of ASCs [52]. The authors demonstrated that YTHDF2 plays a crucial role in regulating the expansion of HSCs in vivo by modulating the stability of various mRNAs essential for HSC self-renewal [52].

METTL14, another major m^6^A methyltransferase, is highly expressed in normal HSPCs and reduced during myeloid differentiation. Inhibition of METTL14 promotes terminal myeloid differentiation of normal HSPCs [53].

Collectively, these results clarify the profound effects of m^6^A in the process of hematopoiesis. METTL3, METTL14, and YTHDF2 may serve as key targets for m^6^A to regulate HSC self-renewal and differentiation in clinical research.

### 4.2. m^6^A and NSCs

m^6^A is critical in regulating neuronal development and adult neurogenesis [54]. Loss of *METTL3* considerably reduces m^6^A levels in adult NSCs (aNSCs) and inhibited the proliferation of aNSCs without affecting their homogeneity [55]. *METTL3* deficiency not only inhibits the development of neurons but also makes the differentiation of aNSCs more inclined toward glial lineage; the morphological maturation of new neurons in the adult brain is also affected [55]. METTL3-mediated m^6^A modification regulates histone methyltransferase Ezh2 expression at the translational level. Ezh2 overexpression could alter neuronal development and neurogenesis defects caused by *METTL3* deficiency [55]. In addition, METTL3 may contribute to spinal cord regeneration. Xing et al. showed a conserved feature of METTL3 changes in a mouse spinal cord injury model, in which the expression of *METTL3* is increased in NSCs [56]. Another m^6^A methyltransferase METTL14 is required for NSC proliferation and maintains NSCs in an undifferentiated state. *METTL14* knockout decreases NSC proliferation and promotes premature NSC differentiation, which suggests that m^6^A is necessary for NSC self-renewal [57].

Similarly, m^6^A demethylase FTO is key in neurodevelopment and neurogenesis. *FTO* is strongly expressed in neurons and aNSCs and dynamically expressed during postnatal neurodevelopment [58]. *FTO* deletion results in reduced brain size and body weight. The lack of *FTO* can inhibit the proliferation and neuronal differentiation of aNSCs in vivo, leading to learning and memory impairment in mice [58]. Cao et al. showed that FTO deficiency in aNSCs transiently increases the proliferation of aNSCs and promotes neuronal differentiation, but in a long term, FTO deficiency inhibits adult neurogenesis and neuronal development through modulating the Pdgfra/Socs5-Stat3 pathway [59] (Figure 2).

The deletion of the m^6^A reader protein YTHDF2 in the embryonic neocortex seriously affects the self-renewal of neural stem and progenitor cells (NSPCs) and the spatiotemporal generation of neurons and other cell types [60]. NSPC proliferation and differentiation ability were significantly reduced in *YTHDF2^−/−^* embryos in both in vivo and in vitro experiments [60]. *YTHDF2^−/−^* neurons cannot produce normal functional neurites, and expression of genes rich in neural development pathways is remarkably interfered [60]. Increased levels of m^6^A modified transcripts are caused by delayed degradation of mRNAs in *YTHDF2^−/−^* NSPCs, which may result in neurogenesis defects [60].

Taken together, the writers, erasers, and readers of m^6^A modification all participate in the development of the nervous system and have crucial effects on neurogenesis.

### 4.3. m^6^A and BMSCs

BMSCs have multidirectional differentiation potential, can support hematopoiesis, promote HSC implantation, participate in key biological processes such as tissue regeneration and immune privilege, and have broad application prospects in stem cell therapy and regenerative medicine [61]. The m^6^A modification is involved in the development and differentiation of BMSCs. The expression of m^6^A methyltransferase METTL3 is significantly upregulated in the adipogenic process of porcine BMSCs (pBMSCs) [62]. A lack of *METTL3* in pBMSCs can promote adipose formation and mediate Janus kinase 1 (*JAK1*) expression in an m^6^A–YTHDF2-dependent manner [62]. *METTL3* deficiency reduces the m^6^A levels of JAK1, thus enhancing the stability of YTHDF2-dependent JAK1 mRNA. JAK1 affects adipogenesis by regulating STAT5 expression and activity. STAT5 can directly bind to the CCAAT/enhancer binding protein (C/EBP) *β* promoter to regulate its activity and mediate JAK1-regulated adipogenic gene expression and thus affect adipogenesis [62] (Figure 2). This study provides a new perspective for the potential molecular mechanism of m^6^A modification in regulating BMSC differentiation into adipocytes and may provide crucial reference values in stem cell regenerative medicine and obesity treatment.

Moreover, *METTL3* deficiency in BMSCs may lead to bone impairment, insufficient osteogenic differentiation ability, and increased adipogenic potential. Yu identified METTL3 as a crucial regulator in the progression of osteogenic differentiation [63]. *METTL3* is highly expressed in osteogenically differentiated BMSCs [64]. Loss of *METTL3* suppressed the osteogenic differentiation potential of BMSCs [64]. Yan et al. reported that METTL3-induced m^6^A methylation of RNAs promotes osteogenic differentiation of BMSCs through m^6^A-based posttranscriptional regulation of runt-related transcription factor 2 (RUNX2) [65]. Silence of *METTL3* by short interfering RNA (siRNA) decreased m^6^A methylation levels and inhibited osteogenic differentiation of BMSCs and reduced bone mass [65]. In addition, *METTL3* overexpression in BMSCs can prevent osteoporosis caused by estrogen deficiency in mice [66]. In terms of mechanisms, the parathyroid hormone (PTH)/parathyroid hormone receptor-1 (PTH1R) signal axis is a pivotal m^6^A downstream pathway in BMSCs [66]. *METTL3* conditional knockout reduces the translation efficiency of PTH1R in BMSCs and interferes with PTH-induced osteogenic and adipogenic effects in vivo [66] (Figure 2). These results provide new insights into the key regulatory role of m^6^A in bone health and disease as well as new evidence for the regulation of stem cell differentiation by m^6^A.

The demethylase activity of FTO is required for MSC differentiation. Wang et al. demonstrated that exposure of MSCs to TNF-*α* is sufficient to repress *FTO* expression, leading to increased *Nanog* mRNA methylation, decreased *Nanog* mRNA expression, and reduced differentiation potential of MSCs [67].

## 5. m^6^A and Induced Pluripotent Stem Cells

**Figure 3:**
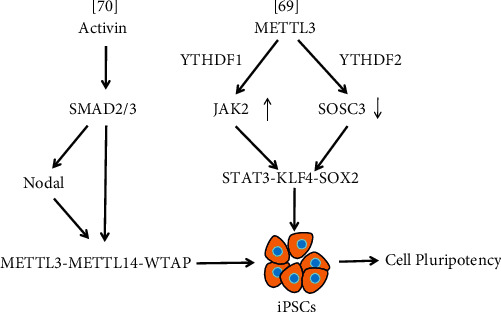
The schematic of interplays between Activin/Nodal and STAT3-KLF4-SOX2 signaling pathway with m^6^A modulators in regulation of iPSC pluripotency.

iPSCs were originally obtained by using viral vectors to transfer four transcription factors, OCT4, SOX2, Kruppel-like factor 4 (KLF4), and c-Myc, into differentiated somatic cells and reprogramming them. iPSCs have broad application prospects in the treatment of human diseases and organ transplantation [68]. Because of their similarity to and physiological characteristics in the human genome, porcine iPSCs (piPSCs) have become an ideal alternative research model to human ESCs (hESCs). The m^6^A modification plays a major role in mediating the pluripotency of piPSCs. *METTL3* deletion remarkably affects cell self-renewal and pluripotency in piPSCs. METTL3 controls the STAT3–KLF4–SOX2 signal pathway by mediating JAK2 and SOSC3 expression in a YTHDF1/YTHDF2-orchestrated manner to regulate piPSC pluripotency [69] (Figure 3, Table 1).

Bertero et al. reported that the intracellular effectors SMAD2 and SMAD3 (SMAD2/3) interact with the METTL3–METTL14–WTAP complex in an Activin/Nodal signal-dependent manner in both hESCs and human iPSCs (hiPSCs) [70] (Figures 1 and 3, Table 1). The interaction could promote m^6^A deposition on transcript subsets participating in early cell fate determination. The resulting negative feedback makes these transcripts unstable and leads to rapid degradation after inhibiting the Activin/Nodal signal [70]. The mechanism facilitates timely withdrawal of pluripotency and induces neuroectodermal differentiation.

The m^6^A reader proteins YTHDF2 and YTHDF3 are required for reprogramming somatic cells into iPSCs [71]. *YTHDF2* is highly expressed in iPSCs and downregulated during neural differentiation [72]. Depletion of *YTHDF2* in iPSCs leads to stabilization of a group of m^6^A-modified transcripts associated with neural development, loss of pluripotency, and induction of neural-specific gene expression [72] (Table 1).

## 6. m^6^A and CSCs

CSCs are a small subset of various tumor types that have the dual characteristics of self-renewal and differentiation and are crucial in tumor occurrence and development. CSCs are resistant to most treatments and are therefore related to cancer recurrence [73]. Therefore, developing a more effective treatment necessitates further exploration of the molecular mechanism of CSC regulation.

### 6.1. m^6^A and LSCs

Compared with healthy HSPCs or other types of cancer cells, m^6^A methyltransferase METTL3 is more abundant in acute myeloid leukemia (AML) cells. In addition, *METTL3* loss in human myeloid leukemia cell lines can lead to cell differentiation and apoptosis and hinder the progress of leukemia in recipient mice in vivo. m^6^A can promote the translation of c-Myc, Bcl-2, and PTEN mRNAs in the human AML MOLM-13 cell line [47]. Barbieri et al. also demonstrated that *METTL3* is a crucial gene for AML cell growth [74]. In immunodeficient mice, downregulation of *METTL3* leads to cell-cycle arrest, leukemia cell differentiation, and leukemia establishment failure. METTL3 is related to chromatin and is located at the transcriptional initiation sites of active genes. Moreover, most of these genes have the CAATT-box binding protein CEBPZ at the transcription initiation site, which is necessary for METTL3 to be recruited into chromatin [74]. Promoter-bound METTL3 induces m^6^A modification in the coding region of relevant mRNA transcripts and improves its translation efficiency by mitigating ribosome stagnation [74]. These results suggest that METTL3 may be a potential target for myeloid malignancy treatment.

METTL14, another m^6^A methyltransferase, is strongly expressed in AML cells carrying t(11q23), t(15;17), or t(8;21) and is reduced during myeloid differentiation [53]. METTL14 inhibition promotes terminal myeloid differentiation of AML cells and inhibits AML cell survival and proliferation. METTL14 is essential for the development and maintenance of AML and self-renewal of leukemia stem initiation cells (LSCs or LICs) [53]. METTL14 plays a carcinogenic role by regulating its key targets, such as *MYB* and *MYC*, through m^6^A modification.

**Figure 4:**
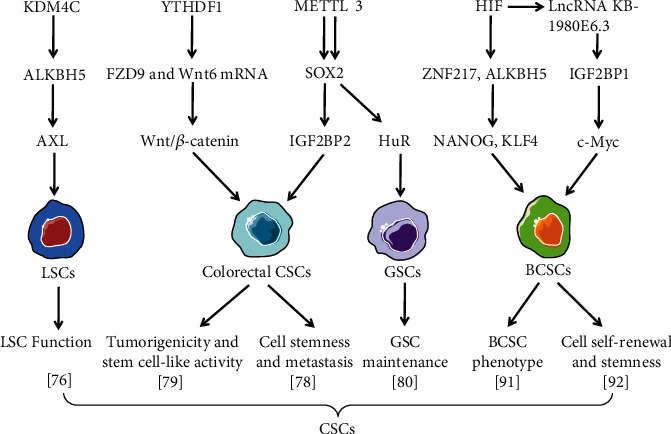
The schematic of interplays between m^6^A modulators and multiple factors in regulation of different CSC phenotypes, tumorigenicity, and metastasis.

The demethylase ALKBH5 plays critical roles in leukemic cell transformation, AML development and maintenance, and LSC/LIC self-renewal through posttranscriptional regulation of critical targets via m^6^A-dependent mechanisms [75]. Shen et al. demonstrated that targeting ALKBH5 effectively inhibits AML development/maintenance and suppresses LSC self-renewal while sparing normal hematopoiesis [75]. Wang et al. showed that ALKBH5 is required for maintaining LSC function but is dispensable for normal hematopoiesis and reveal KDM4C-ALKBH5-AXL signaling axis in AML development and maintenance [76] (Figure 4). These findings suggest a potential therapeutic strategy for selectively treating AML by targeting ALKBH5.

The m^6^A reader protein YTHDF2 has been shown to be overexpressed in a broad spectrum of human AML [77]. YTHDF2 is essential for disease initiation and reproduction in mouse and human AML. YTHDF2 can reduce the half-life of m^6^A transcripts, which are crucial for the overall integrity of LSC function [77]. Notably, the authors found that *YTHDF2* is not required for normal HSC function and that *YTHDF2* deletion may increase HSC activity [77].

### 6.2. m^6^A and CRC CSCs

One study revealed the basic functions of m^6^A in colorectal carcinoma (CRC) and demonstrated the carcinogenic effects of METTL3 in promoting stemness and metastasis of CRC. Colorectal cancer stem-like cells are a cancer cell type with self-renewal and multiple differentiation potential and have strong tumorigenicity and metastasis abilities. METTL3 inhibition could enhance chemotherapy response and reduce CSCs in CRC [78]. METTL3 maintains the expression of the CSC marker SRY-box 2 (SOX2) through the m^6^A–IGF2BP2-dependent regulatory mechanism in CRC cells and then promotes CRC stemness and metastasis [78] (Figure 4). In addition, METTL3 inhibition could reduce the surface antigen expression of colorectal CSCs. Overall, these results highlight the critical role of METTL3 in CRC, indicating that METTL3 may be a CSC marker for CRC diagnosis and treatment.

The m^6^A reader protein YTHDF1 is involved in tumorigenesis. YTHDF1 knockdown considerably inhibited the tumorigenicity of CRC cells in vitro and the growth of mouse xenograft tumors in vivo [79]. *YTHDF1* silencing reduced the number of colonospheres, downregulated CRC CSC marker expression, and inhibited Wnt/*β*-catenin pathway activity by interacting with FZD9 and Wnt6 mRNA in CRC cells [79] (Figure 4). These results indicate that YTHDF1 is key in tumorigenicity and stem cell-like activity in CRC cells and may provide a new potential target for the clinical treatment of CRC.

### 6.3. m^6^A and GSCs

The m^6^A mRNA modification plays a key role in glioblastoma stem cell (GSC) self-renewal and tumorigenesis. Visvanathan et al. reported that METTL3-mediated m^6^A modification plays a key role in GSC maintenance and glioma cell dedifferentiation [80]. *METTL3* expression is elevated in GSC and attenuated during differentiation. The METTL3-dependent GSC maintenance is mediated by SOX2 mRNA stabilization, and human antigen R (HuR) recruitment to m^6^A-modified sites is required for SOX2 mRNA stabilization [80] (Figure 4). *METTL3*-silenced GSCs exhibited increased sensitivity to *γ*-irradiation and decreased DNA repair. Exogenous overexpression of 3′UTR-less SOX2 exhibited potent DNA repair in *METTL3*-silenced GSCs [80]. In addition, METTL3 is essential for the expression of GSC-specific actively transcribed genes [81]. The integrated analysis of the m^6^A regulome in *METTL3*-silenced GSCs showed global disruption in tumorigenic pathways that are indispensable for GSC maintenance and glioma progression [81]. Li et al. identified METTL3 as a modulator of nonsense-mediated mRNA decay to sustain malignancy in glioblastoma (GBM) [82]. Silencing *METTL3* or overexpressing dominant-negative mutant *METTL3* suppressed the growth and self-renewal of GSCs [82]. These data suggest that METTL3 may be a molecular target for clinical GBM treatment. Moreover, *METTL3* or *METTL14* deletion can significantly enhance GSC growth, self-renewal, and tumor progression [83]. By contrast, *METTL3* overexpression or *FTO* suppression can inhibit GSC growth and self-renewal. In addition, FTO inhibition can suppress tumor progression and prolong the lifespan of GSC-transplanted mice [83]. Further analysis demonstrated that m^6^A modification participates in regulating the expression of genes with crucial biological functions in GSCs, which may be a target for GBM treatment.

The levels of m^6^A demethylase ALKBH5 are high in GSCs. ALKBH5 is necessary for GSC self-renewal, and increased *ALKBH5* expression typically indicates a poor prognosis in patients with glioblastoma (GBM), whereas *ALKBH5* deletion can inhibit GSC proliferation and tumorigenesis [84]. Kowalski-Chauvel et al. demonstrated that targeting ALKBH5 increases radiosensitization of GSCs by controlling the homologous repair and represses their invasion capability [85]. These data suggested that ALKBH5 is an attractive therapeutic target to overcome radioresistance and invasiveness of GSCs. Huff et al. showed that FTO inhibitor FTO-04 can impair the self-renewal properties of GSCs to prevent neurosphere formation without significantly altering the growth of human NSC neurospheres [86].

Yarmishyn et al. demonstrated that YTHDF1 is involved in Musashi-1-mediated GBM tumorigenesis processes such as cell proliferation and migration and also regulates the stem-like properties of GBM cells [87]. YTHDF2 was identified as a GSC-specific dependency that regulates glucose metabolism in GSCs through stabilization of MYC transcripts [88].

### 6.4. m^6^A and BCSCs

The breast cancer stem cells (BCSCs) are the crucial factors for the occurrence, growth, metastasis, and recurrence of breast cancer [89]. BCSC phenotype is specified and maintained by the expression of octamer-binding transcription factor 4 (OCT4), Kruppel-like factor 4 (KLF4), SRY-box 2 (SOX2), and NANOG [90]. Hypoxia can promote BCSC enrichment in breast cancer cells, which depends on the activity of hypoxia-inducible factor (HIF) [90]. Knockout of ZNF217 or ALKBH5 gene can increase m^6^A RNA methylation and decrease the levels of NANOG and KLF4 under hypoxic conditions [91]. The expressions of hypoxia-induced pluripotent factors and ALKBH5 or ZNF217 in breast cancer cell lines are dependent on HIF. The expression of HIF-1*α* and ALKBH5 were consistent in human breast cancer biopsies analyzed. Knockout of ALKBH5 in MDA-MB-231 breast cancer cells remarkably reduced breast-to-lung metastasis in immunodeficient mice [91] (Figure 4). This study suggests that m^6^A demethylase ALKBH5 and m^6^A methyltransferase inhibitor ZNF217 have important effects on BCSC phenotype and breast cancer metastasis. Zhu et al. reported that hypoxia-induced lncRNA KB-1980E6.3 is involved in the self-renewal and stemness maintenance of BCSCs by recruiting IGF2BP1 to regulate c-Myc mRNA stability. The lncRNA KB-1980E6.3/IGF2BP1/c-Myc axis may potentially be a therapeutic target for breast cancer [92] (Figure 4).

Overall, these results indicate that m^6^A modification has an important influence on the occurrence and development of various cancers and may be a potential target for cancer treatment (Table 1).

## 7. m^5^C and Stem Cells

m^5^C is another crucial posttranscriptional RNA modification. However, studies related to this modification and stem cell biology are limited.

An unbiased global analysis of total RNA and nuclear poly(A) RNA m^5^C in mouse ESCs and mouse brains indicated that m^5^C loci accumulated considerably near the codon of translation initiation, depleted from the m^6^A peak region of the translation stop codon, and increased at different locations in 3′UTRs in different transcript classes [93]. This study provides a comprehensive map of cytosine methylation in the transcriptome of murine pluripotent and differentiation stages and provides crucial reference values for future studies of the biological function of m^5^C in mammalian RNA. The RNA m^5^C methyltransferase NSUN3 regulates ESC differentiation by affecting mitochondrial activity. *NSUN3* mutant cells exhibited significant reductions in mt-tRNA^Met^ methylation and formylation as well as mitochondrial translation and respiration [94]. Although the proliferation of *NSUN3* mutant cells decreased, pluripotency marker gene expression was not affected [94]. ESC differentiation had a tendency toward mesoderm and endoderm lineages at the expense of neuroectoderm [94]. Thus, these findings demonstrate that m^5^C RNA modification plays a crucial role in regulating ESC fate and function (Table 1).

m^5^C RNA methylation is involved in the regulation of NSC differentiation and motility. The m^5^C methyltransferase NSUN2 is expressed in neuroepithelial stem and progenitor cells during early human brain development, and its expression gradually decreases during neural differentiation [95]. Deletion of NSUN2-mediated tRNA methylation increases their endonucleolytic cleavage by angiogenin, resulting in the enrichment of 5′-derived tRNA fragments in *NSUN2−/−* brains [95]. *NSUN2* depletion inhibits the migration of neural cells to chemical attractant fibroblast growth factor 2, leading to impaired neural differentiation of neuroepithelial stem cells [95] (Table 1). These findings indicate that m^5^C RNA methylation has crucial effects on neural development, and its role in other stem cells requires further exploration and research.

## 8. U-Tail and Stem Cells

U-tail is closely related to stem cell differentiation and cancer (Table 1). Zcchc11 (TUT4) was identified as the 3′ TUTase responsible for Lin28-mediated pre–let-7 U-tail and blockade of let-7 processing in mouse ESCs [96]. Zcchc6 (TUT7) was also identified as an alternative TUTase that functions with Lin28 in vitro [97]. Zcchc11 and Zcchc6 redundantly control let-7 biogenesis in ESCs [97]. These results provide insight into the mechanism of Lin28-mediated TUTase control of let-7 expression in stem cells and cancer. In addition, Takahashi et al. reported that TUT7 is involved in the neural differentiation of primed pluripotent stem cells via the regulation of human endogenous retrovirus accumulation [98].

DIS3L2 is a 3′–5′ exonuclease responsible for the decay of uridylated let-7 precursor in mouse ESCs [99, 100]. Pirouz et al. showed that DIS3L2 is required for normal ESC differentiation [101]. DIS3L2 deficiency resulted in the formation of larger embryoid bodies during spontaneous ESC differentiation [101]. Liu et al. found that an lncRNA (AC105461.1), a promoter upstream transcript of DIS3L2, may be a mediator of CRC stem cells [102]. The expression of AC105461.1 was positively correlated with that of DIS3L2 in CRC [102]. AC105461.1 overexpression impaired the CSC properties while knockdown enhanced the CSC properties, including self-renewal, migration, and invasion abilities [102]. The role of U-tail in various stem cells cannot be ignored, and it is worth more exploration in the future.

## 9. Conclusions and Perspectives

RNA methylation, as a vital posttranscriptional mechanism of gene regulation, is crucial to physiological and pathological processes. As high-throughput sequencing technology is rapidly developing, the understanding of the biological functions of RNA methylation is deepening.

Numerous studies demonstrated a close correlation between RNA methylation and stem cells. RNA methylation plays a key role in regulating stem cell maintenance, differentiation, reprogramming, and controlling in mammalian developmental stages. The regulatory factors of RNA methylation are involved in regulating stem cell fate and function. These findings have opened new directions for the clinical treatment of various diseases, including cancer. The aberrant expression of one or more RNA methylation regulatory factors may be used as a diagnostic or prognostic biomarker. Further in-depth analysis of RNA methylation may aid in developing inhibitors targeting writers, erasers, or readers to further explore the potential mechanisms for controlling gene expression in stem cells in physiology and pathology. However, many obstacles remain that must be overcome when treating diseases by targeting RNA methylation. In addition, although RNA methylation involves many patterns, research on RNA methylation patterns in addition to m^6^A in stem cells is limited and may require further exploration and research as well as more advanced molecular biology techniques to make breakthroughs.

## Figures and Tables

**Table 1:** RNA methylation regulators and their functions in different types of stem cells.

Stem cell types	RNA methylation	Writer	Eraser	Reader	Related function	Reference
Embryonic stem cells (ESCs)	m^6^A	METTL14			ESC stemness and differentiation	[35]
	m^6^A m^6^A m^6^A	METTL3 METTL3, WTAP METTL5			Naive pluripotency ESC differentiation ESC pluripotency and differentiation	[36] [37] [12, 38]
	m^6^A	WTAP-Virilizer-Hakai			ESC self-renewal and differentiation	[39]
	m^6^A	METTL3-METTL14-WTAP			Early cell fate determination and pluripotency	[70]
	m^6^A		FTO		ESC pluripotency	[40]
	m^6^A			YTHDF3	ESC pluripotency	[41]
	m^6^A	METTL3		YTHDC1	Chromatin accessibility and transcription	[42]
	m^6^A			YTHDF1/2/3	ESC differentiation	[43]
	m^5^C	NSUN3			ESC proliferation and differentiation	[94]
	U-tail			DIS3L2	ESC differentiation	[101]

Hematopoietic stem cells (HSCs)	m^6^A	METTL3			Cell differentiation and cell proliferation Endothelial-to-hematopoietic transition The maintenance of HSC identity and symmetric commitment	[47, 49–51]
	m^6^A			YTHDF2	Endothelial-to-hematopoietic transition HSC self-renewal	[51, 52]
	m^6^A	METTL14			Terminal myeloid differentiation of normal HSPCs	[53]

Neural stem cells (NSCs)	m^6^A	METTL3			Proliferation and differentiation of adult NSCs Neuronal development and neurogenesis	[55]
	m^6^A	METTL14			NSC proliferation and differentiation	[57]
	m^6^A		FTO		Proliferation and neuronal differentiation of adult NSCs	[58, 59]
	m^6^A			YTHDF2	Self-renewal, proliferation, and differentiation of NSCs	[60]
	m^5^C	NSUN2			Migration and differentiation of neuroepithelial stem and progenitor cells	[95]

Bone marrow mesenchymal stem cells (BMSCs)	m^6^A	METTL3			BMSC differentiation	[62–66]
	m^6^A		FTO		MSC differentiation	[67]
	m^6^A			YTHDF2	Adipogenesis	[62]

Induced pluripotent stem cells (iPSCs)	m^6^A	METTL3		YTHDF1/YTHDF2	Self-renewal and pluripotency of iPSCs	[69, 72]
	m^6^A	METTL3-METTL14-WTAP			Early cell fate determination and pluripotency	[70]
				YTHDF2 YTHDF3	iPSC reprogramming	[71]
	U-tail	TUT7			The neural differentiation of primed PSCs	[98]

Cancer stem cells (CSCs)	m^6^A	METTL3			Myeloid leukemia cell differentiation and apoptosis Maintenance of the leukemic state Colorectal CSC stemness phenotype and metastasis of CRC GSC maintenance and dedifferentiation of glioma cells GSC growth, self-renewal, and tumor progression	[47, 74, 78, 80–83]
	m^6^A	METTL14			Development and maintenance of AML self-renewal of leukemia stem and initiation cells GSC growth, self-renewal, and tumor progression	[53, 83]
	m^6^A			YTHDF2	The overall integrity of LSC function GSC growth	[77, 88]
	m^6^A			YTHDF1	CRC CSC marker expression GBM tumorigenesis	[79, 87]
	m^6^A		ALKBH5		LSC self-renewal and function Self-renewal, proliferation, and tumorigenesis of GSCs Radioresistance and invasiveness of GSCs BCSC phenotypes and breast cancer metastasis	[75, 76, 84, 85, 91]
	m^6^A		FTO		GSC growth, self-renewal, and tumor progression	[83, 86]
	m^6^A			IGF2BP2	CRC stemness and metastasis	[78]
				IGF2BP1	Self-renewal and stemness maintenance of BCSCs	[92]
	U-tail			DIS3L2	CSC properties	[102]
